# A New Polymeric Hybrid Auxetic Structure Additively Manufactured by Fused Filament Fabrication 3D Printing: Machine Learning-Based Energy Absorption Prediction and Optimization

**DOI:** 10.3390/polym16243565

**Published:** 2024-12-20

**Authors:** Rezgar Hasanzadeh

**Affiliations:** Department of Mechanical Engineering, Kermanshah University of Technology, Kermanshah 6715685420, Iran; r.hasanzadeh@kut.ac.ir

**Keywords:** polymers, auxetic, 3D printing, machine learning, negative Poisson’s ratio

## Abstract

The significance of this paper is an investigation into the design, development, and optimization of a new polymeric hybrid auxetic structure by additive manufacturing (AM). This work will introduce an innovative class of polymeric hybrid auxetic structure by the integration of an arrow-head unit cell into a missing rib unit cell, which will be fabricated using fused filament fabrication (FFF) technique, that is, one subset of AM. The auxetic performance of the structure is validated through the measurement of its negative Poisson’s ratio, confirming its potential for enhanced energy absorption. A chain of regression, linear, and quadratic polynomial machine learning algorithms are used to predict and optimize the energy absorption capability at variant processing conditions. Amongst them, the polynomial regression model stands out with an R-squared value of 92.46%, reflecting an excellent predictive capability for energy absorption of additively manufactured polymeric hybrid auxetic structure. The optimization technique revealed that the printing speed of 80 mm/s and layer height of 200 µm were the critical values to achieve a maximum amount of energy absorption at 5.954 kJ/m^2^, achieved at a printing temperature of 244.65 °C. Such results also contribute to the development of AM, since they show not only the potential for energy absorption of polymeric hybrid auxetic structures but also how effective machine learning is in the optimization of the AM process.

## 1. Introduction

Additive manufacturing is also more famously known as 3D printing and has opened a whole new dimension in material science with respect to object conception, design, and manufacture. From humble and modest beginnings as a method to perform rapid prototyping of simple structures, this process has evolved into an advanced technique through which one can create structures of extreme intricacy with great precision and speed [1,2]. Among the plenty of different materials that emerged in this new era, the auxetic structure stands out for its unusual property to become thicker perpendicularly while stretched due to a negative Poisson’s ratio [3,4]. So unique is this characteristic that it opens a completely new world in the field of material engineering. The introduction of fused filament fabrication (FFF) has played a huge role in the development of additive manufacturing capabilities and continues to lead today. FFF enables the production of intricate geometries that were once deemed unachievable, allowing for the exploration of complex auxetic designs that can be tailored to specific applications [5].

Polymeric hybrid auxetic structures have come as a huge leap in engineering innovation. These are conceptualized from an elaborate integration of different kinds of unit cells, each of which attributes to the overall auxetic behavior derived in this regard [6]. The strategic integration of these cells is bound to yield a hybrid structure with greatly advanced properties in mechanics and flexibility. Applications for these structures range from aerospace to biomedical engineering, where they have many emerging uses in specialty materials with superior mechanical properties [7]. In materials science terms, this means energy absorption capability in auxetic structures is an issue of prime interest. Such materials are theoretically supposed to have better energy dissipation due to their unique deformation mechanics [8]. Therefore, the auxetic structures, especially their hybrid design, would be able to boast this property and present themselves as an ideal candidate in applications requiring resistance to high impacts or considerable damping of vibration [9,10].

The possibility of improving the mechanical characteristics of auxetics, by Bagewadi et al. [11], was explored through a novel hybrid structure development. The hybrid structure was realized using both honeycomb and re-entrant unit cells. First, they additively manufactured the cellular structures, then conducted the study on uniaxial quasi-static compression of auxetic and hybrid auxetic structures loaded in different directions. These findings prove that hybrid geometry structure may be realized with higher specific energy absorption and compressive strength than the usual auxetic structure. Chang et al. [12] investigated the auxetic property of the structure with rotating square and proposes an elastic base material with an intention to increase strength yet still retain auxetic behavior. Modulus difference between materials in creating the auxetic effect has been emphasized. This approach consisted of the modeling, by finite element analysis, of auxetic behavior in a 2-layer laminated system, discussing the influence brought about by different values of modulus between the frame and base material. Mojaver et al. [13] discussed new design and testing of auxetic structure made of thermoplastic polyurethane using 3D printing technology. In this context, they experimentally produced new auxetic structures by 3D printing of thermoplastic polyurethane samples and numerically compared the measured Poisson’s ratios with simulated results obtained by Ansys 2022 R1 software.

Mahesh et al. [14] have presented an experimental investigation into auxetic structures manufactured from polymer nanocomposite materials for compressive behavior, negative Poisson’s ratio, and energy absorption characteristics evaluated in terms of parameters such as peak crushing strength, negative Poisson’s ratio, specific energy absorption, and crush force efficiency. The fabrication of auxetic structures by FFF technique was performed. Higher loading of the nanoclay particles reduced the compressive strength of the structures. The negative Poisson’s ratio decreases with an increase in the rate of strains; composition, stiffness, and geometrical parameters affect strain energy absorption. Khadem-Reza et al. [15] presented the design of 3D structures that possessed negative Poisson’s ratio for energy absorption, utilizing S-shaped auxetic unit cells. Simulation and experimental testing validated the structures for energy-absorbing system applications. The designed S-shaped auxetic unit cells were printed using 3D printing technology and exhibited negative Poisson’s ratio properties in two perpendicular planes. Mazur and Shishkovsky [16] proposed the combination of analytical modeling, numerical simulations, and experimental testing in investigating the properties and behavior of auxetic materials, including their hierarchical structures. Multi Jet 3D printing of anisotropic lattices was used for mechanical testing. Conclusions focused on the fact that hierarchical auxetic materials bear unprecedented potential to be precisely tuned for prescribed mechanical characteristics across various applications.

Machine-learning algorithms have brought much change in predictive analysis for material behavior. These algorithms include most simple-like linear regressions to more complex quadratic and polynomial regressions, offering new approaches toward the performance prediction of materials under different conditions [17,18]. The coefficient of determination—popularly known as R-squared—states the proportion of variability within the dependent response variable explained by the independent parameter(s) and has often been used to choose between competing algorithms. Optimization of hybrid auxetic structures for energy absorption via machine learning algorithms is not only a present challenge but also a very promising line of investigation. By harnessing the predictive power of such algorithms, refinements in designing auxetic structures are possible and might, someday, also revolutionize yet another approach to material design [19]. Further advances can be expected in this continuously evolving field that will push the boundaries of possibilities with auxetic materials.

Recent research has been directed at applications of machine learning in FFF optimization. Investigation on the effects of printing parameters on mechanical properties and surface quality has been performed with methodologies such as classification and regression trees for predicting the optimum settings for composite structures [20]. The development of machine learning models, including support vector regression, has also utilized sensor data from 3D printers in several instances to predict both extrusion force and mechanical properties [21]. Machine learning has also coupled with finite element analysis in relating experimental and predicted values of Young’s modulus [22]. Further, several hyperparameter tuning techniques, such as grid search, have been used with multilayer perceptron models in optimizing their performance for the prediction of FFF outcomes [23]. These studies show the capability of machine learning in improving FFF processes through the prediction and optimization of several features concerning print quality and mechanical properties.

The relevance of this study is that, for the first time, it introduces a new design for auxetic structures using AM. In this project, the arrow-head unit cell and the missing rib unit cell have been integrated to introduce a novel polymeric hybrid auxetic structure with the potential to push forward the boundaries of material flexibility and energy absorption. Application of the FFF for the fabrication of such a structure, not only was a proof of potentiality of various AM techniques, but also it acted as a testimony to the most complex geometries among the most elaborative designs. Characterization of Poisson’s ratio of the structure in detail along with further verification of the auxetic features inside it, speaks volume regarding viability along with efficiency of the design. Optimizing energy absorption using machine learning algorithms is a completely new approach and can be referred to as bigamy between material science and data analytics. The contributions hereby made are manifold, varying from presenting a new perspective on the capability of AM, enriching the understanding of auxetic materials, to setting up a comparative framework-a robust framework for the prediction and improvement of manufactured structure energy absorption. This research not only paves the way for future advancements in the field but also establishes a benchmark for the integration of computational methods in material design and analysis.

## 2. Materials and Methods

### 2.1. Machine Learning Algorithms

Artificial intelligence has ushered in a newer era in material sciences, and machine learning (ML) algorithms apparently are leading from the front this technological revolution [24,25]. Within additive manufacturing, ML algorithms have started being used increasingly to interpret complex datasets and forecast material behavior with an unprecedented degree of accuracy [26]. These algorithms are of paramount importance, as they help optimize production processes, adding value to the final product and shortening the period dedicated to research and development processes [27]. Machine learning algorithms develop predictive capabilities for relationships in data that cannot be described from conventional analytics for insight into the performance of manufactured structures [28].

Therein, this work will serve as a proof of the capability for performance prediction related to auxetic structures using linear, quadratic, and polynomial ML algorithms, as follows:

(1) $$\upsilon =x_{1}A+x_{2}B+x_{3}C+x_{0}$$

(2) $$\upsilon =x_{1}A+x_{2}B+x_{3}C+x_{4}A^{2}+x_{5}B^{2}+x_{6}C^{2}+x_{0}$$

(3) $$\upsilon =x_{1}A+x_{2}B+x_{3}C+x_{4}A^{2}+x_{5}B^{2}+x_{6}C^{2}+x_{7}AB+x_{8}AC+x_{9}BC+x_{0}$$

where υ denotes Poisson’s ratio, *A*, *B*, and *C* are the processing conditions, and $x_{i}$ shows the coefficients.

These will add significantly to the literature by allowing the investigation of the effects of different printing parameters on the energy absorption capability of the polymeric hybrid auxetic structure. On the other hand, one could take a linear model to reach a somewhat straightforward approach, the quadratic model allows capturing the basic curvature in relationships of data. The most noticeable work is that the use of polynomial models, capable of fitting even complex and nonlinear interactions, has contributed to the fine-tuning of the manufacturing process for optimal energy absorption [29].

Regression machine learning analysis is a powerful tool in data analysis to predict continuous outcomes based on input variables [30]. The fitting of the regression model to data enables the identification of patterns among data variables, the institution of relations to produce accurate forecasting and decision making. This technique finds its application in many industries [31,32]. With the rapid availability of data, coupled with an improvement in machine learning algorithms, regression analysis now finds immense relevance in extracting insights from complex, continuously coming datasets [33].

The methodology in conducting a regression analysis typically includes the following [34,35]:

- Collect data on relevant variables: Include both predictor variables, also known as independent variables, and the outcome variable (also called the dependent variable).
- Data preprocessing: Cleaning the data, which involves the handling of missing values, removal of outliers, and encoding of categorical variables.
- Data split: A split in the data is carried out into training and testing sets to evaluate the performance of the model.
- Model selection: That would be the selection of an appropriate regression model considering data nature as well as the problem statement. Common regression models include linear regression, polynomial regression, and multiple regression.
- Model training: The chosen regression model is fitted on the training data to learn the relationships between predictor variables and an outcome variable.
- Model evaluation: Perform model evaluation of the trained model with metrics like mean squared error, R-squared, and adjusted R-squared.
- Model tuning: Fine-tune the model by adjusting hyperparameters to improve its predictive accuracy.
- Prediction: Use the trained regression model to make predictions on new data or test data.
- Interpretation: Interpret the coefficients obtained from the regression model, to understand how each of the predictor variables plays a part in influencing the outcome variable.

### 2.2. Experimental Procedure

#### 2.2.1. Hybrid Auxetic Structure Design

**Figure 1:**
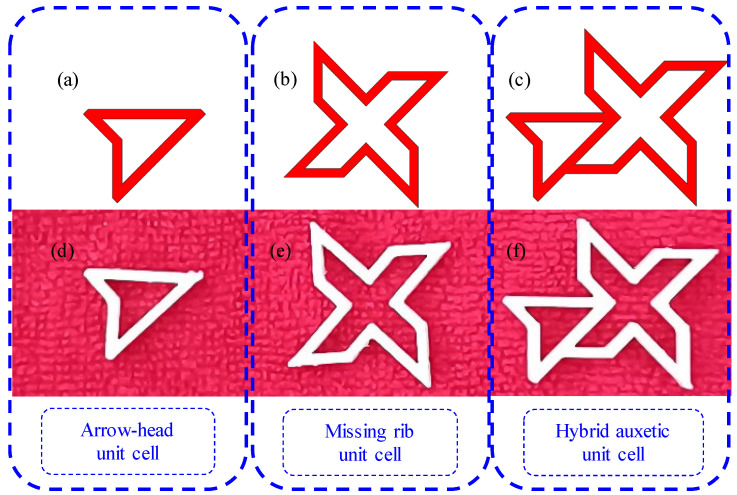
Basic auxetic unit cells and developed hybrid auxetic unit cell; (**a**,**d**) show the arrow-head unit cell design and additively manufactured unit cell; (**b**,**e**) show the missing rib cell design and additively manufactured unit cell; (**c**,**f**) show the developed hybrid auxetic unit cell design and additively manufactured unit cell.

In this paper, a polymeric hybrid auxetic structure is proposed by combining an arrow-head unit cell with a missing rib unit cell. The designed and additively manufactured arrow-head unit cell is represented in Figure 1a,d. Figure 1b and Figure 1e represent the additively manufactured and designed unit cells of the missing rib, respectively. Figure 1c and Figure 1f show, respectively, the designed and additively manufactured hybrid auxetic unit cell—as manufactured.

**Figure 2:**
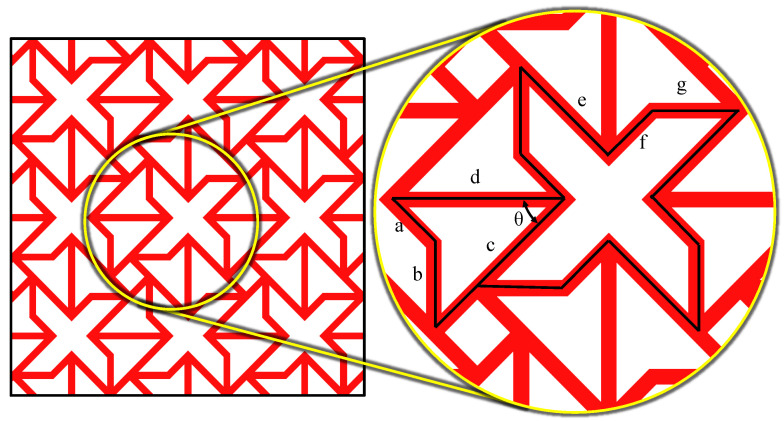
Hybrid auxetic structure and the structural dimensions of the hybrid auxetic unit cell; a = 5 mm, b = 7 mm, c = 15 mm, d = 15 mm, e = 10 mm, f = 5 mm, g = 8 mm, and θ = 45°.

Figure 2 presents the hybrid auxetic structure, including the dimensions of the concerned structure in this work. To enable an effective study of the processing parameters and their influence on the efficiency of the structure, all the dimensional parameters in the structure are kept constant. In the arrow-head unit cell, there are two base sides, *a* and *b*, respectively, which measure 5 mm and 7 mm. It involves two height sides, referred to as *c* and *d* and has a length of 15 mm. The angle *θ* between the vertical surfaces is 45°. On the contrary, the missing rib unit cell involves three elements comprising *e*, *f*, and *g* whose lengths are 10 mm, 5 mm, and 8 mm, respectively.

#### 2.2.2. Additive Manufacturing of Auxetic Structures

The CREALITY-CR 10-Smart 3D printer, Shenzhen, China, has been used in the current study for the fabrication of polymeric hybrid auxetic structures.

Different materials used in sample preparation for this study are: Thermoplastic polyurethane (TPU); due to its outstanding flexibility, it is used in samples to measure Poisson’s ratio. Samples to undergo tests related to energy absorption are prepared with polyethylene terephthalate glycol (PETG) because of its remarkable toughness. The e-TPU-95A filament supplied from eSUN, Esun Industrial Co., Ltd., Shenzhen, China, was used with a density of 1.21 g/cm^3^, and a melt flow index of 1.2 g/10 min at 190 °C/2.16 kg. The same goes for the filament PETG supplied by eSUN, Esun Industrial Co., Ltd., Shenzhen, China, but having a density of 1.27 g/cm^3^ and having a melt flow index of 20 g/10 min at 190 °C/2.16 kg.

In the case of samples with TPU, printing parameters were carefully set at printing temperature = 215 °C; printing speed = 25 mm/s; heat bed temperature = 60 °C; infill density = 100%; number of walls = four walls; layer height = 200 µm, nozzle diameter = 400 µm. For printing the PETG samples, the heat bed temperature is maintained at 75 °C. On the other hand, all the other parameters such as infill density, wall count, and nozzle diameter remain constant as that for TPU samples. Printing temperature, speed, and layer height emerge as key variable inputs; they shall be used in determining the effect on the performance of the polymeric hybrid auxetic structure and in the development of machine learning algorithms, as described in Table 1.

#### 2.2.3. Trials Design

The estimate of the effectiveness of the polymeric hybrid auxetic structure is based on the analysis of three basic processing parameters in the 3D printing environment, namely, printing temperature, printing speed, and layer height. These three parameters have been used as basic inputs for the development of machine learning algorithms with the purpose of foreseeing performance in such a structure. The experimental design is developed based on a Box–Behnken design where each of these parameters has been stratified along three different levels. The Box–Behnken design optimally selects parameters with the help of a three-level, fractional factorial design. It requires a central composite structure for which each factor must be studied at three levels, namely low, medium, and high. Trials must be designed in such a way that all possible combinations of factors at their respective levels are included, excluding the extreme corners to increase the efficiency of the experiment. The Box–Behnken design has been considered one of the most efficient methods of experimental design, particularly in cases where fewer numbers of trials are required compared with another design [36]. This method is better for multivariate systems; it can provide optimal conditions with minimal runs of experiments [37]. In addition, the Box–Behnken design has the advantage in estimation of main effects, interactions of the factors, and quadratic terms without the need for a full factorial design [38]. This approach therefore guarantees that this study comprehensively explores the effects of processing parameters on the performance of a polymeric hybrid auxetic structure while ensuring economical and methodical application of resources. The levels of the 3D printing process parameters used in printing are the temperature of 220 °C, 240 °C, and 260 °C; the printing speed of 20 mm/s, 50 mm/s, and 80 mm/s; and the layer height of 120 µm, 160 µm, and 200 µm. These parameters and their levels are systematically cataloged in Table 2. The experimental trials have been arranged according to the Box–Behnken design; the detailed layout is given in Table 3. It should be noted that all the statistical analysis were implemented in the Minitab software version 18.

#### 2.2.4. Performance Evaluation

**Figure 3:**
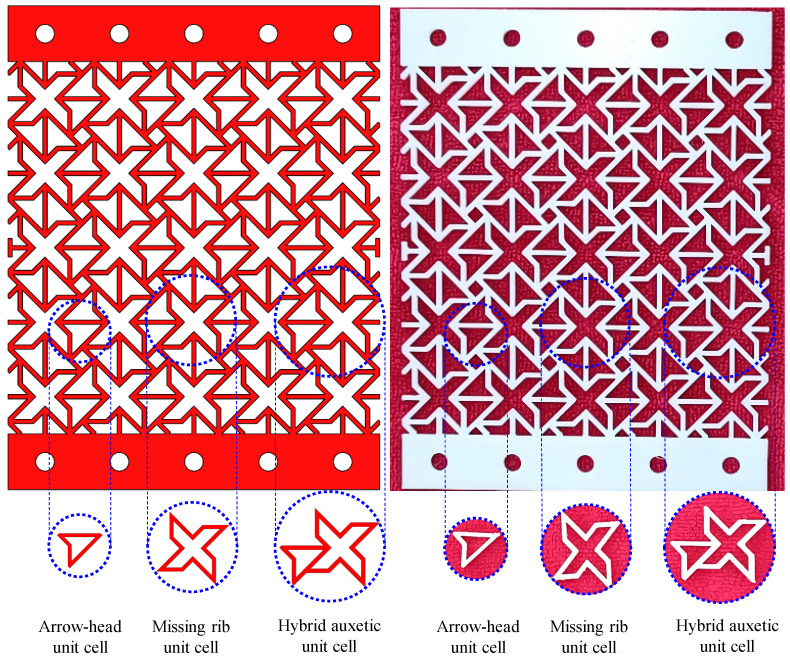
Designed and additively manufactured TPU samples using polymeric hybrid auxetic structure for the Poisson’s ratio test.

The performance evaluation of the polymeric hybrid auxetic structure includes Poisson’s ratio and energy absorption. Under conditions where there was a variation in strain, Poisson’s ratio was measured to approve the auxetic nature of the structure. As illustrated in Figure 3, a representative sample was fabricated using TPU filament with a dimension of 10 × 10 cm^2^, representing the hybrid auxetic unit cell. The precision measurements were performed with a digital caliper, series 1108, INSIZE Co, Ltd., Derio, Spain, built on an accuracy of 0.01 mm. At least three samples received each strain, after which the average value was recorded as the definitive output.

**Figure 4:**
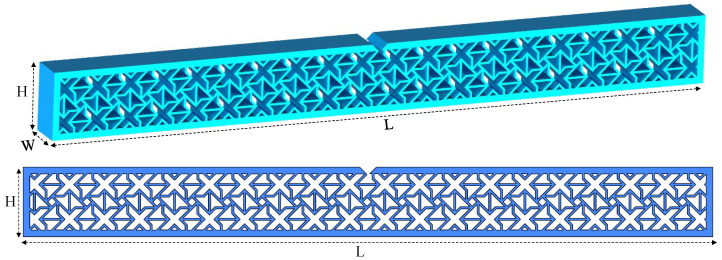
Designed samples using hybrid auxetic structure for energy absorption test based on the ASTM D6110 standard (L = 125 mm, H = 12.7 mm, and W = 7 mm).

Energy absorption performance was studied. The ASTM D6110 standard was followed for specimen design, as shown in Figure 4. Specimens were additively manufactured using PETG filament. Application of this two-faceted methodology for Poisson’s ratio and energy absorption will provide comprehensive understanding of the mechanical properties of the polymeric hybrid auxetic structure.

The capabilities of absorption of energy within fabricated polymeric hybrid auxetic structure samples were investigated with the help of a Charpy impact testing method on a Noavaran Baspar Co., Tehran, Iran, Charpy impact testing apparatus. Energy absorption was measured based on the amount of dissipated energy before failure, using a ratio to the cross-sectional area at the fracture point. The equation governing the calculation of energy absorption, obtained from [39], is given below:

(4) $$E_{a}\;(\frac{J}{m^{2}})=\frac{(cos\beta_{2}-cos\beta_{1})Wx}{A}$$

where *W* denotes the mass of the pendulum hammer, *x* shows the hammer length, and $\beta_{1}$ and $\beta_{2}$ are the initial and final angles of the hammer, respectively. The parameters were set with the pendulum length at 0.3948 m, the hammer’s mass at 2.036 kg, and the hammer’s initial angle at 150°. Testing at least three test specimens was carried out in all cases to obtain a statistically relevant mean for the absorbed energy as the final measure of impact strength.

**Figure 5:**
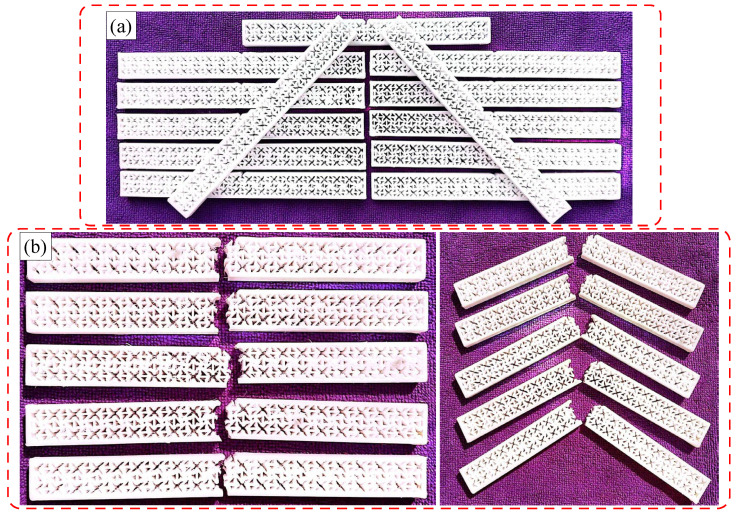
Representative additively manufactured PETG samples using polymeric hybrid auxetic structure for energy absorption test based on the ASTM D6110 standard; (**a**) shows the samples before the test and (**b**) shows the fractured samples after the test.

Figure 5 presents a graphical comparison in the case of PETG hybrid auxetic samples before and after Charpy impact tests to capture the material behavior in response to dynamic loading conditions.

## 3. Results and Discussion

### 3.1. Analysis of Auxetic Structure

**Figure 6:**
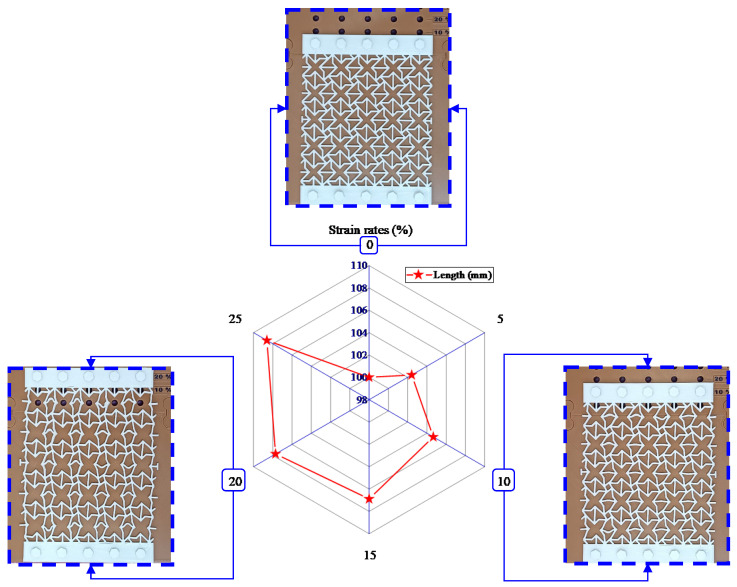
Results of width changes in the polymeric hybrid auxetic structure at various strains.

Figure 6 shows the change in dimensions of polymeric additively manufactured samples with hybrid auxetic structure under stretching after different strains expressed through 0%, 5%, 10%, 15%, 20, and 25%. Also, some of the samples are visually presented in the same figure which, after additive manufacturing, show polymeric hybrid auxetic structures in their deformed state. Poisson’s ratios were calculated with due care to realize the auxetic characteristics of the material after the width of the samples was quantified post-deformation.

**Figure 7:**
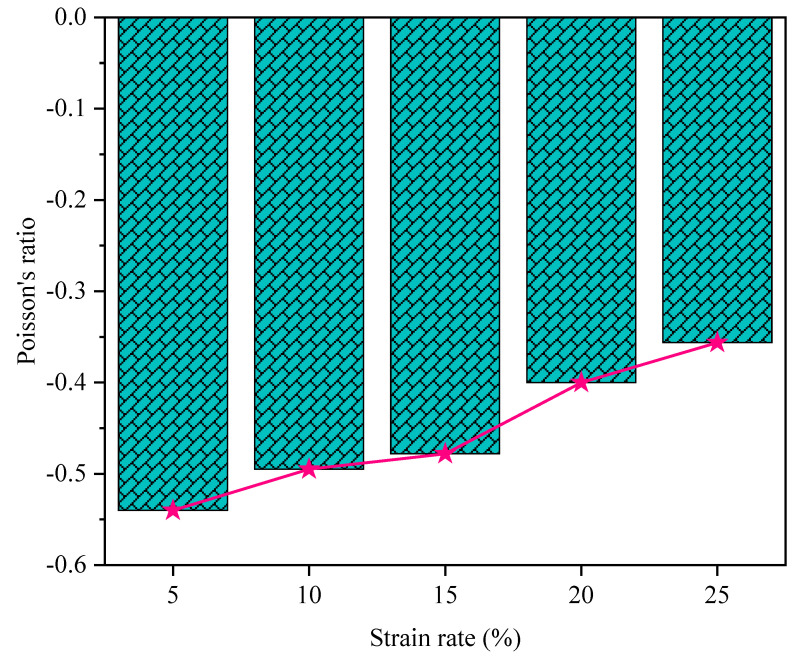
Results of Poisson’s ratio of the polymeric hybrid auxetic structure at various strains.

Figure 7 presents the experimental results of Poisson’s ratios obtained for the additively manufactured polymeric hybrid auxetic structure subjected to different strains. The data confirm the achievement of negative Poisson’s ratios for all the tested strains, confirming the auxetic characteristic of the polymeric hybrid structure designed in this work. The highest value of the negative Poisson’s ratio was determined at −0.540 at a strain of 5%. Beyond that, the magnitude of the negative Poisson’s ratio decreased with increasing strains: −0.495 at 10%, −0.478 at 15%, −0.400 at 20%, and −0.356 at 25% strains.

### 3.2. Developing Machine Learning Algorithms

In implementing the feature of optimizing energy absorption capability, three different ML algorithms-linear, quadratic, and polynomial-have been developed in this paper for additively manufactured polymeric hybrid auxetic structures. These algorithms are built based on the obtained empirical results from energy absorption tests besides taking printing temperature, *A*, printing speed, *B*, and layer height, *C*, as the main input variables as follows:

(5) υ=−0.0007A+0.02015B+0.00429C+2.05

(6) $$\upsilon =0.276A-0.0143B-0.0520C-0.000577A^{2}+0.000344B^{2}+0.000176C^{2}-26.0$$

(7) $$\upsilon =0.231A-0.0626B-0.1782C-0.000577A^{2}+0.000344B^{2}+0.000176C^{2}-0.000291AB+0.000372AC+0.000739BC-9.3$$

**Figure 8:**
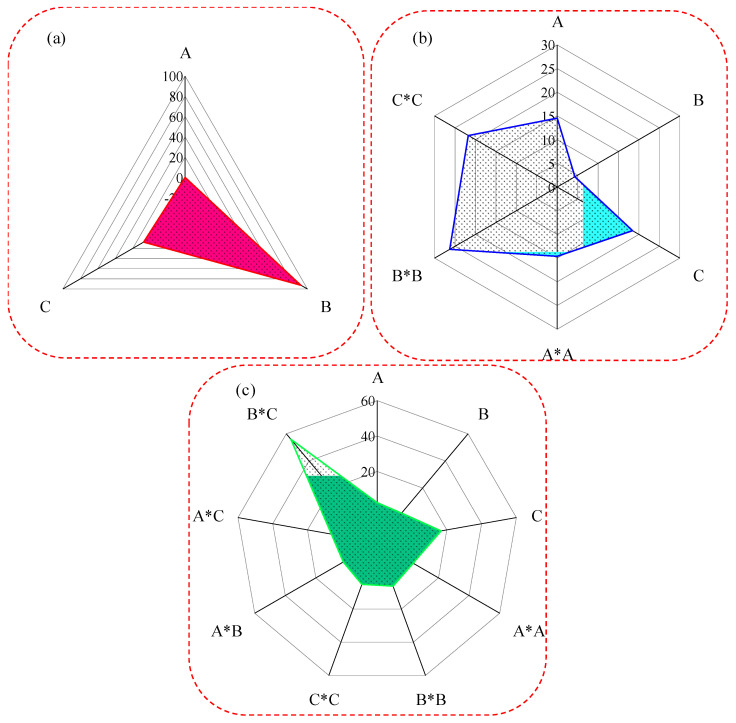
Contributions of parameters to the machine learning algorithms of (**a**) linear machine learning algorithm, (**b**) quadratic machine learning algorithm, and (**c**) polynomial machine learning algorithm (A is the linear term of temperature, B is the linear term of printing speed, C is the linear term of layer height, A*A is the quadratic term of temperature, B*B is the quadratic term of printing speed, C*C is the quadratic term of layer height, A*B is the interaction term of temperature and printing speed, A*C is the interaction term of temperature and layer height, and B*C is the interaction term of printing speed and layer height).

Figure 8 depicts the correlation of the mentioned input variables with the performance of the ML algorithms using the analysis of variance. Figure 8a shows that the linear ML algorithm is highly dependent upon the printing speed, which alone contributes 92.50% to its predictive power, while the layer height has a rather modest contribution of 7.45%. the printing temperature seems to have no noticeable effect since its contribution stands at 0.05%. Figure 8b gives an insight into the quadratic ML algorithm, showing that its quadratic terms are highly influential when compared to its linear terms. The quadratic terms of printing speed and layer height, each contributing up to 26.30% and 21.76%, respectively, are highly influential in this algorithm. Another highly influential term is the linear term for layer height, contributing 18.44%. For the temperature of printing, both the linear and quadratic terms are almost equally influential, showing a contribution of just over 14% from each. The linear term in printing speed is not very dominant, with a contribution of 4.33%, yet it is part of the quadratic algorithm. In the polynomial ML algorithm, as shown in Figure 8c, the interaction term of printing speed with the layer height becomes the most dominant contributor with a contribution capability of 55.99%. The linear term of layer height becomes the second most contributing factor to the prediction capability of the algorithm with a contribution capability of 16.70%. While the rest of the terms are individually below 7%, their combined contribution is not negligible; their importance reflects the complexity of the polynomial algorithm and thus indicates that it captures complex relationships among the data.

**Figure 9:**
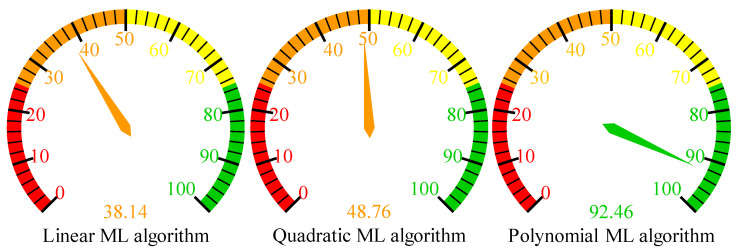
Results of R-squared values for different machine learning algorithms highlighting the outstanding performance of polynomial machine learning algorithm.

The accuracy in the ML algorithms for predictive modeling is fundamental. There has been an R-squared value considered as an excellent ranking metric for variance estimation within the dependent variables, which can be predictable from the independent variables [40,41]. Figure 9 shows that the R-squared value of the linear ML algorithm has only 38.14%, which seems to be relatively low within this study. This may point out the inability of a linear model to capture such relationships, probably because the relationships between the processing parameters and energy absorption performance of the additively manufactured polymeric hybrid auxetic structure are not linear. While the quadratic ML algorithm shows some moderate improvement in R-squared, with a value of 48.76%, as described by Figure 9, it is still far from correct prediction. It might be considered an insufficiency of the quadratic model, given that the quadratic terms probably did not capture the complex interaction and higher-order relationships within the data. By sharp contrast, the polynomial ML algorithm returns an R-squared of 92.46%, indicating a highly accurate model. One of the critical implications of such superior performance is that the algorithm can model a complex and multi-dimensional data landscape, capturing the nuanced interplay of input variables with energy absorption outcomes.

Its high R-squared value, when compared to the other two ML algorithms, reflects the robustness and preciseness of a polynomial model in predicting energy absorption in a polymeric hybrid auxetic structure. Linear and quadratic models gave low R-squared values, indicating inability in handling the multi-faceted nature of the data. It can, therefore, be said that among the evaluated models, the polynomial algorithm is the most reliable due to its adeptness in handling nonlinear patterns and interactions.

**Figure 10:**
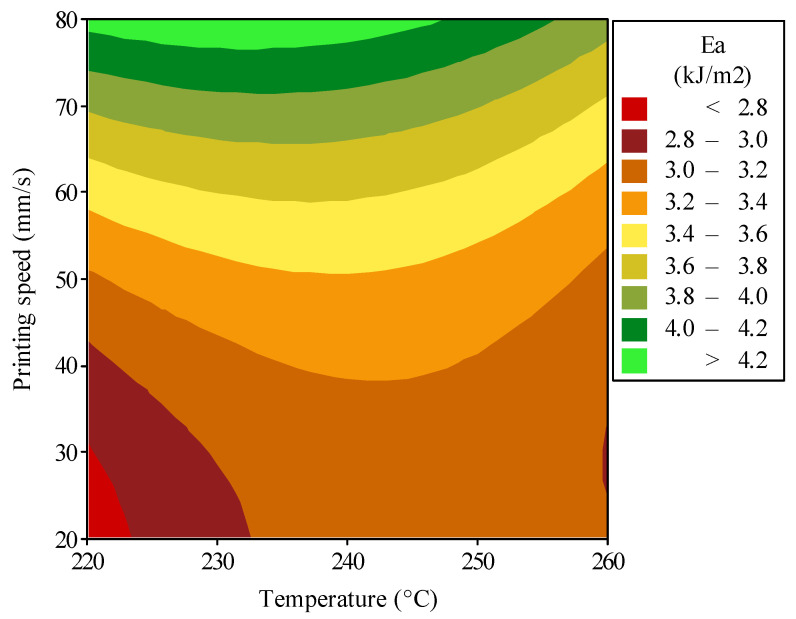
Optimization results to enhance the energy absorption performance of the polymeric hybrid auxetic structure by simultaneous controlling printing temperature and speed.

Figure 10 presents the contour plot that is one of the main graphical representations obtained by the optimization study, showing the energy absorption of the additively manufactured polymeric hybrid auxetic structure versus two of the most important processing parameters: printing temperature and printing speed. The temperature range runs from 220 to 260 °C and the printing speed ranges from 20 to 80 mm/s. It clearly showed that the contour areas vary dramatically in energy absorption. This graphic is instrumental in determining the set of processing conditions that yields maximum energy absorption by the structure. The contour plot suggests that printing speed has a higher influence on energy absorption than printing temperature. Increasing printing speed will greatly enhance the absorption of energy. This could be because at higher extrusion rates there would be additional layer bonding and reduced porosity, thus giving a more cohesive material structure which can dissipate the energy more effectively. On the other hand, the influence of printing temperature is subtler: there is just a little more energy absorbed by increasing printing temperature at the lower speeds, probably because of better melt flow at increased temperatures, allowing for improved adhesion between layers up to some limit beyond which any benefit starts to become saturated. The contour plot shows optimization insights where peak energy absorption, approximately 4.2 kJ/m^2^, should occur at a printing speed higher than 78 mm/s and a printing temperature below 250 °C. Such conditions may create an optimum balance between material flow and rapid solidification that constructs a structure of good strength. On the contrary, energy absorption becomes minimum when printing speed is less than 30 mm/s and temperature is below 223 °C. Such unfavorable conditions may result in an insufficient fusing of interlayers, which leads to poor material properties and a reduction in the capability of the structure for absorbing energy.

**Figure 11:**
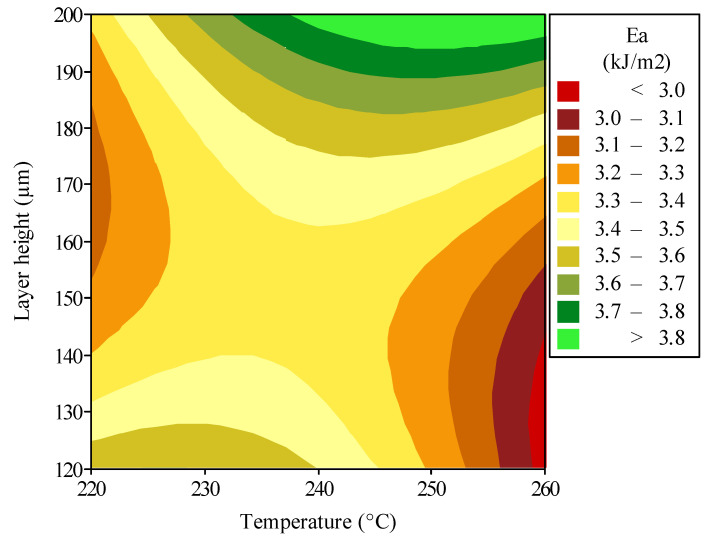
Optimization results to enhance the energy absorption performance of the polymeric hybrid auxetic structure by simultaneous controlling printing temperature and layer height.

Figure 11 shows the optimization for energy absorption of the polymeric auxetic structure in a contour plot which maps the interplay of printing temperature and layer height on energy absorption of the additively manufactured polymeric auxetic structure. These contours, falling within the temperatures of 220–260 °C and layer heights of 120–200 µm, form a plot that guides towards the regions of peak performance in parameter space. It is realized that although the layer height effect on energy absorption is significant, there exists a corresponding increase in energy dissipation capability with the increment in layer height, more so at the highest printing temperature. This could be indicative that higher layer heights may allow for the development of a stronger interlayer adhesion, especially when the material is deposited at a temperature that optimizes flow and solidification dynamics. Contrary to that, the effect of printing temperature caused bifurcation at the lower layer heights, whereas an increase in temperature tends to lessen energy absorption at minimal layer height, it enhances absorption at much greater layer heights. The reason for this dichotomy might relate to the thermal dynamics involved in the printing process: a temperature that is too high can lead to material degradation at thin layers but improve fusion and toughness at thicker layers. This corresponds to approximately 3.8 kJ/m^2^ energy absorption at the apex, under conditions where the layer height is greater than 195 µm and the printing temperature is higher than 237 °C. Such parameters may create an optimal environment for forming a dense and cohesive material structure, therefore optimizing the inherent energy-dissipating characteristics of the auxetic design. In contrast, for a layer height less than 140 µm at a temperature over 258 °C, the minimum energy absorption is nearly at 3.0 kJ/m^2^. This might be an indication that, under such conditions, the material may not achieve structural integrity sufficient to attain optimal energy absorption because of probably poor layer bonding or thermal degradation.

**Figure 12:**
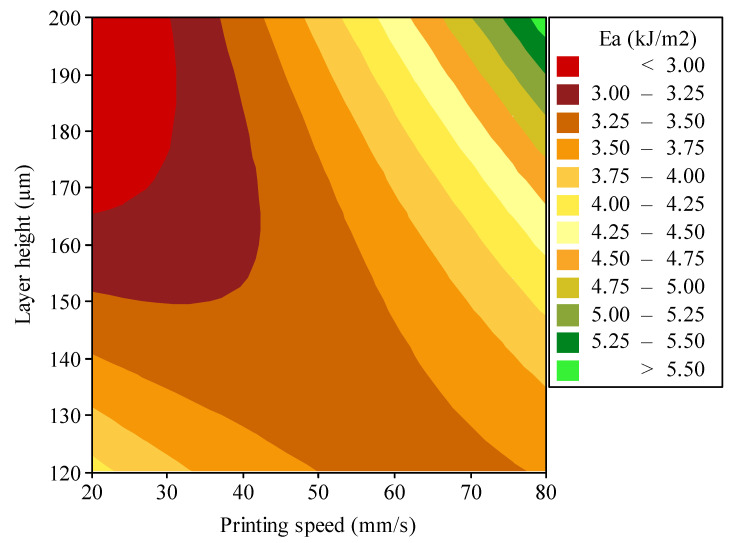
Optimization results to enhance the energy absorption performance of the polymeric hybrid auxetic structure by simultaneous controlling printing speed and layer height.

Figure 12 shows the combined effects of printing speed and layer height on the energy absorption of the additively manufactured polymeric auxetic structure. Gradations from 20 to 80 mm/s of printing speed and 120 to 200 µm of layer height carry, in visual form, the energy absorption spectrum of this structure, which shows the optimal manufacturing conditions. Energy absorption increases substantially with the increment of layer height and becomes even stronger at higher printing speeds. This may be due to the fact that larger layers would lead to a much stronger bond between successive strata, in particular when deposited at speed, thereby giving the material greater capability to resist and dissipate impact energy. At the same time, better energy absorption, higher at increased layer heights, is attributed to an increase in the printing speed; this may be due to the reduced cooling time, which enables even more adequate adiabatic adhesion between layers and the homogeneous deposition of the material. The peak of energy absorption is estimated at 5.50 kJ/m^2^; this may provide a very optimum thermal and mechanical environment for the auxetic structure to realize its full potential in terms of energy dissipation. In this case, printing speed is at its maximum, as is the layer height. On the other extreme, the lowest energy absorption occurs at approximately 3.0 kJ/m^2^ for layer heights higher than 165 µm, coupled with printing speeds below 32 mm/s. At such low speeds, sufficient heat cannot be supplied to ensure good adhesion of interlayers for higher thicknesses of layers, hence a weakening of energy absorption in the structure.

**Figure 13:**
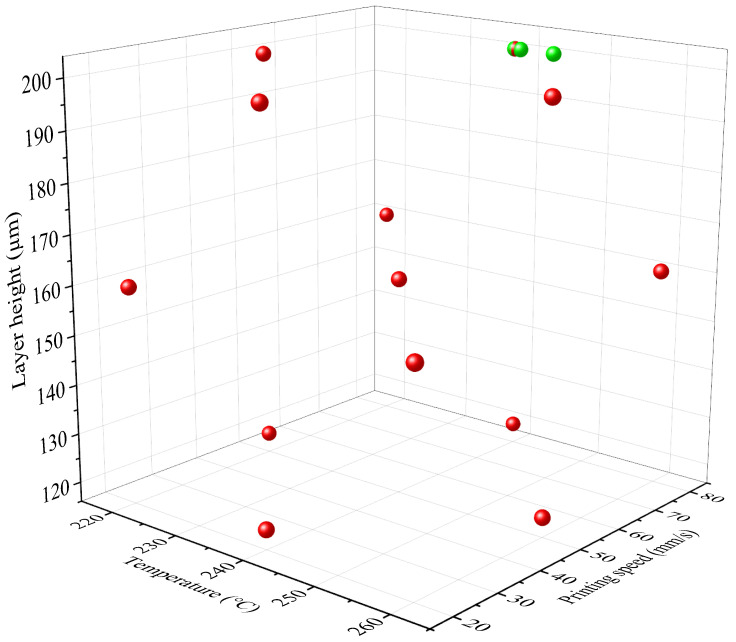
Optimization of processing parameters to achieve the maximum energy absorption performance of the polymeric hybrid auxetic structure (red circles show the processing conditions and green circles specify the optimum solutions).

Figure 13 shows the optimum processing parameters where the additively manufactured polymeric auxetic structure absorbs the maximum amount of energy. These optimum conditions were obtained using the polynomial machine learning algorithm in the Minitab software. The outputs of the polynomial ML algorithm are four optimal points in the parameter space that converge to 80 mm/s printing speed and 200 µm layer height, but differ in printing temperature: 240 °C, 239.19 °C, 244.65 °C, and 239.18 °C. These are the coordinates of points in parameter space where the structure’s energy absorption capabilities are maximized according to the predictive model developed by the algorithm. From a detailed analysis, these optimal conditions tend towards higher printing speeds and layer heights, meaning these factors are important in improving the energy-absorbing capability of the material. The changes in printing temperature, though within a small range, show the fine balance between possessing enough thermal energy for the material to flow and the layers to bond and not destroying the integrity of the material [42,43]. If the printing temperatures are too low, the filament will not be suitably melted, and between layers, adhesion will not be good. Different voids and weak points are developed between layers; because of that, there is a loss in structural integrity of the sample. Due to partial melting, the deposition of layers is not uniform. This also decreases the capacity of energy absorption of the samples further. On the other hand, a too high printing temperature causes thermal degradation in the polymeric material. Excessive heat can degrade the polymer chains that eventually alter the mechanical properties, toughness, and strength of the material [44]. High temperatures increase warping and deformation likelihood in the printed parts, which can have a negative influence on their energy absorption performance. At the optimum printing temperature, the filament melts adequately; this ensures good interlayer adhesion and uniform layer deposition.

The desirability analysis further refines the method for selecting optimized conditions, and the set of parameters—printing temperature of 244.65 °C, printing speed of 80 mm/s, and layer height of 200 µm—gave a desirability value of 90.75%. This not only outperforms all other identified points but also returns an impressive energy absorption value of 5.954 kJ/m^2^. Thus, this result touts the superior performance of this particular set of conditions, probably because the interplay of thermal input and deposition rate is at an optimal position where the creation of a structure with robustness, yet capable of exceptional energy dissipation, can be realized.

Although much has been achieved by this study in the state of the art in AM, especially in the optimum design of polymeric hybrid auxetic structures for energy absorption, one must declare several limitations: Firstly, the fact that the present study is conducted using the FFF technique, part of the family of AM techniques, could limit generalization to other variants of the AM techniques. In addition, high predictability in the machine learning models depends on a finite dataset and does not reflect all possible variants of conditions for AM and material behaviors. Mechanical testing focused on energy absorption and Poisson’s ratio, possibly at the expense of other key mechanical properties like tensile strength, durability, and long-term stability. Second, the tests were conducted in a lab and might not be perfectly representative of crisscross conditions in manufacturing on an industrial scale. Third, the optimization process was limited to the range of trial parameters evaluated, and although the polynomial regression model provided a very robust prediction within this range, it may not accurately extrapolate behaviors beyond this range.

Building on the results of this present study, several further research opportunities may arise. Other AM techniques than FFF might give a wider overview of how the manufacturing method can alter mechanical properties of polymeric hybrid auxetic structures. It may also be relevant to investigate a wider range of material types, including composites and metals, that will shed further light on the versatility and applicability of the design principles established here. Further integration of other mechanical tests, such as resistance to bending and fatigue life, will give a holistic performance profile that one can expect from this structure. The diversities of auxetic structures’ possible applications in biomedical devices, space, and automotive industries are very exciting. Translation of these research findings into products and technologies requires collaboration by the researcher, industry expert, and policy maker.

## 4. Conclusions

The present work has seen the design, development, and optimization of a novel polymeric hybrid auxetic structure, leveraged on the capability of additive manufacturing (AM). Therefore, such unique integration of the arrow-head unit cell and missing rib unit cell is possible owing to the precision and flexibility of fused filament fabrication (FFF) for the structure noted with its auxetic properties. The negative Poisson’s ratio has been experimentally verified regarding its possible auxetic behavior and high energy absorption, one of the main features for novel materials in several diverse industrial sectors. Machine learning algorithms also played an important role in the prediction and optimization of energy absorption performance of the structure for varying AM processing conditions. Amongst these, the polynomial regression algorithm showed quite excellent accuracy with an R-squared value of 92.46%, revealing quite high accuracy for predictions and, furthermore, proving to be of great value in the guidance of the AM process. The optimization analysis, through the basis of its contour plots and desirability analysis, identified critical processing parameters making a real difference in the energy absorption capabilities of the polymeric auxetic structure. The optimum energy absorption was determined as 5.954 kJ/m^2^ under printing temperature 244.65° C by rigorous optimization with printing speed 80 mm/s and layer height 200 µm. This way, the research contributes to the knowledge base in the AM field by showing the complementary nature of computational modeling with physical experimentation. Interdisciplinary approaches to research that effectively integrate design, materials science, and computational analytics are at the heart of advancing the frontiers of AM technologies. These findings not only provide an avenue for the development of material with superior mechanical properties but also detail a roadmap toward the systematic optimization of the AM process, further reinforcing the transformative effect of AM in modern manufacturing.

## Figures and Tables

**Table 1:** 3D printing processing conditions for sample production.

Parameters	TPU	PETG
Printing temperature (°C)	215	Variable
Printing speed (mm/s)	25	Variable
Heat bed temperature (°C)	60	75
Infill density (%)	100	100
Wall count	4	4
Layer height (µm)	200	Variable
Nozzle diameter (µm)	400	400

**Table 2:** Input variables and their levels for performance evaluation of the polymeric hybrid auxetic structure and development of machine learning algorithms.

Input Variable	Levels		
	Low	Medium	High
Printing temperature (°C)	220	240	260
Printing speed (mm/s)	20	50	80
Layer height (µm)	120	160	200

**Table 3:** Designed trials using the Box–Behnken design technique for performance evaluation of the polymeric hybrid auxetic structure and development of machine learning algorithms.

Run	Temperature (°C)	Printing Speed (mm/s)	Layer Height (µm)
1	220	20	160
2	220	80	160
3	260	50	120
4	260	80	160
5	260	20	160
6	240	50	160
7	240	50	160
8	240	20	200
9	240	80	120
10	240	50	160
11	240	20	120
12	240	80	200
13	260	50	200
14	220	50	120
15	220	50	200

## Data Availability

The original contributions presented in this study are included in the article. Further inquiries can be directed to the corresponding author.
